# Identification and characterisation of enteroaggregative *Escherichia coli* subtypes associated with human disease

**DOI:** 10.1038/s41598-020-64424-3

**Published:** 2020-05-04

**Authors:** Samuel J. Ellis, Lisa C. Crossman, Conor J. McGrath, Marie A. Chattaway, Johanna M. Hölken, Bernard Brett, Leah Bundy, Gemma L. Kay, John Wain, Stephanie Schüller

**Affiliations:** 1 Norwich Medical School, University of East Anglia, Norwich, UK; 2 Quadram Institute Bioscience, Norwich, UK; 3 School of Biological Sciences, University of East Anglia, Norwich, UK; 4 SequenceAnalysis.co.uk, Norwich Research Park, Norwich, UK; 5 Gastrointestinal Bacteria Reference Unit, Public Health England, London, UK; 6 Department of Gastroenterology, Norfolk and Norwich University Hospital, Norwich, UK

**Keywords:** Bacterial pathogenesis, Bacterial genetics, Pathogens, Diarrhoea

## Abstract

Enteroaggregative *E. coli* (EAEC) are a major cause of diarrhoea worldwide. Due to their heterogeneity and carriage in healthy individuals, identification of diagnostic virulence markers for pathogenic strains has been difficult. In this study, we have determined phenotypic and genotypic differences between EAEC strains of sequence types (STs) epidemiologically associated with asymptomatic carriage (ST31) and diarrhoeal disease (ST40). ST40 strains demonstrated significantly enhanced intestinal adherence, biofilm formation, and pro-inflammatory interleukin-8 secretion compared with ST31 isolates. This was independent of whether strains were derived from diarrhoea patients or healthy controls. Whole genome sequencing revealed differences in putative virulence genes encoding aggregative adherence fimbriae, *E. coli* common pilus, flagellin and EAEC heat-stable enterotoxin 1. Our results indicate that ST40 strains have a higher intrinsic potential of human pathogenesis due to a specific combination of virulence-related factors which promote host cell colonization and inflammation. These findings may contribute to the development of genotypic and/or phenotypic markers for EAEC strains of high virulence.

## Introduction

Enteroaggregative *E. coli* (EAEC) are emerging pathogens most commonly associated with acute and persistent paediatric diarrhoea and growth retardation in developing nations^1^. In addition, EAEC are a major cause of acute diarrhoea in travellers to developing countries^2^ and persistent enteric infection in HIV/AIDS patients^3^. Notably, a large outbreak in Germany in 2011 was caused by a hybrid O104:H4 EAEC strain which carried the Shiga toxin gene from enterohaemorrhagic *E. coli* and resulted in over 4,300 cases of diarrhoea, 900 hospitalisations and 50 deaths^4^. EAEC are a heterogenous group and have been traditionally defined by their characteristic “stacked brick”-like aggregative adherence (AA) to HEp-2 cells^5^. Pathogenesis involves adherence to the intestinal epithelium, stimulation of mucus secretion, biofilm formation, cytotoxic damage and mucosal inflammation. Several putative virulence factors involved in these processes have been identified^6,7^. These include the aggregative adherence fimbriae (AAF) of which five major variants have been described so far (AAF/I to AAF/V)^8–12^. AAF are encoded on the pAA virulence plasmid which also contains the major transcriptional regulator AggR and the surface protein dispersin which contributes to AAF dispersal^13–15^. Notably, alternative adhesins such as the *E. coli* common pilus (ECP) can mediate aggregative adherence, particularly in the absence of AAF^16^. Further potential virulence factors of EAEC include the cytotoxins Pet, enteroaggregative *Escherichia coli* heat-stable enterotoxin 1 (EAST-1), and haemolysin E (HlyE) which promote epithelial damage and intestinal colonization^6^. In addition, the serine protease Pic exhibits mucinolytic activity and also stimulates intestinal mucus secretion^17,18^. Despite the identification of multiple putative EAEC virulence candidates, not all EAEC strains harbouring these factors cause human disease, and genotypic studies have failed to consistently associate a single gene or combination of genes with EAEC virulence^6,19^. To determine if certain EAEC lineages were more strongly associated with disease than others, Chattaway and colleagues analysed 564 EAEC isolates from three case-control studies in Bangladesh, Nigeria and the UK by Multi Locus Sequence Typing (MLST)^20^. Of the 17 sequence types (STs) identified, ST40 and ST31 were significantly associated with disease and asymptomatic carriage, respectively. Here, we investigated if the association of EAEC ST40 with disease was linked to specific virulence phenotypes *in vitro*. To this aim, we selected eight strains each of ST40 (diarrhoea-associated) and ST31 (carriage-associated) and determined aggregative adherence (AA) to intestinal epithelium, interaction with mucus, biofilm formation, cytotoxicity and stimulation of an inflammatory response. In addition, all strains were genome-sequenced, and bioinformatic analysis was used to identify putative virulence genes associated with disease potential.

## Results

### ST40 strains adhere better to human colonic epithelium than ST31 isolates

**Figure 1:**
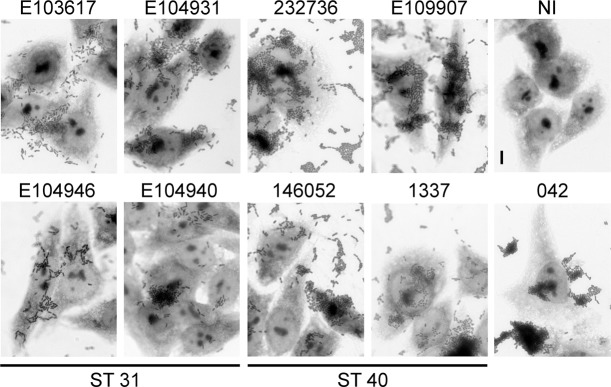
Aggregative adherence of EAEC to HEp-2 cells. Cells were infected with EAEC or left non-infected (NI) for 3 h, and adherent bacteria were visualised by Giemsa staining. Shown are representative images of two experiments performed in duplicate. Bar = 5 μm.

**Figure 2:**
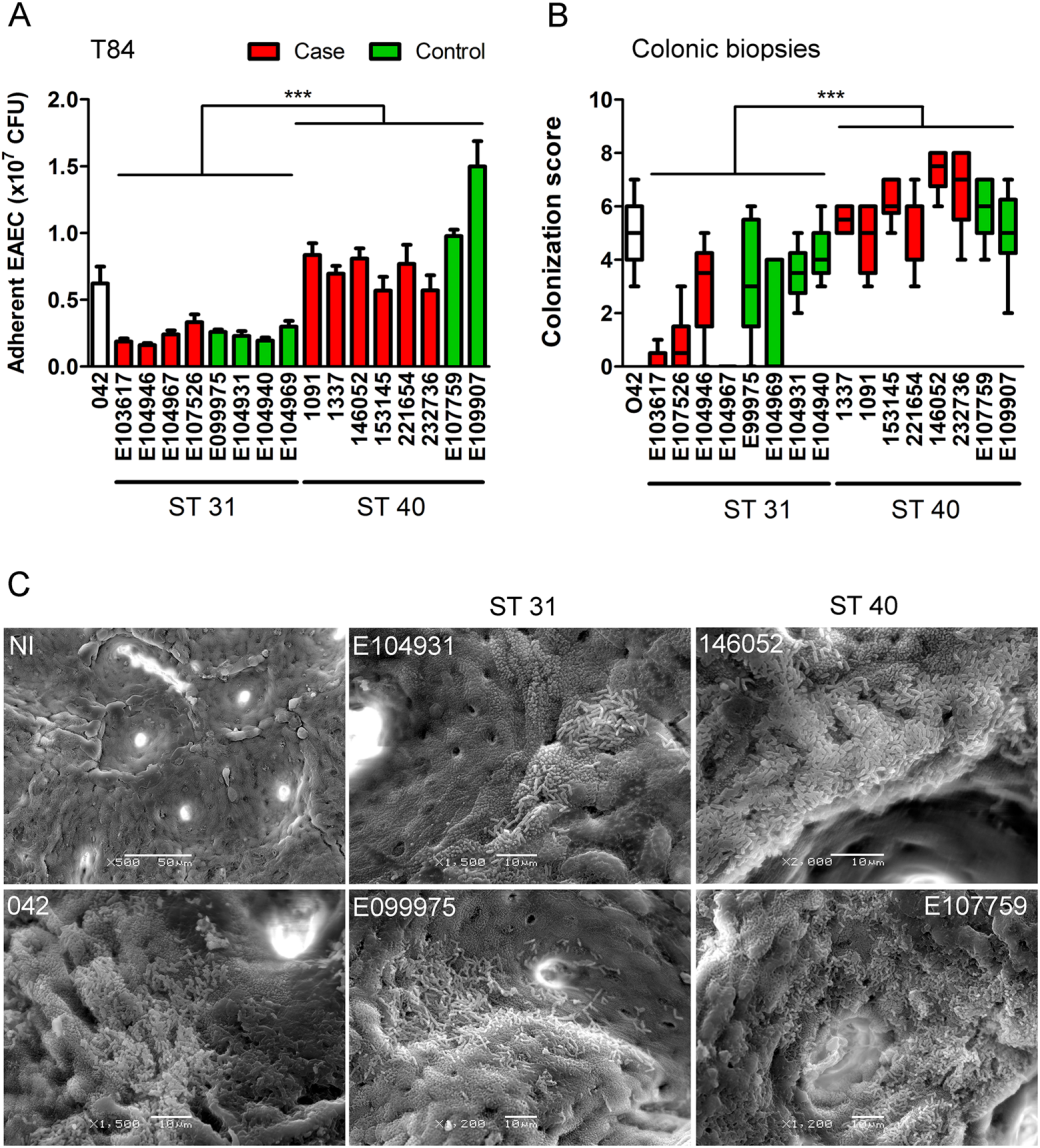
(**A**) Adhesion of EAEC ST strains to T84 cells after 2 h of infection. Adherent bacteria were quantified by determining colony-forming units (CFU) in cell lysates. Results represent the mean ± standard error of the mean (SE) from three independent experiments in duplicate. Data from groups of ST31 and ST40 isolates were analysed using Student’s unpaired t-test. P*** < 0.001. (**B**) Colonization of colonic biopsies after 7 h of incubation. Samples were evaluated by scanning electron microscopy, and colonization was ranked according to the size and frequency of bacterial aggregates as described in methods. Results from three independent experiments in duplicate are shown as box plots with medians. Significance was calculated using the non-parametric Mann-Whitney test. P*** < 0.001. (**C**) Scanning electron micrographs of colonic biopsies infected with EAEC or left non-infected (NI). Shown are representative images of three experiments performed in duplicate. Bar = 10 μm (EAEC), 50 μm (NI).

For this study, eight strains from each ST31 and ST40 were selected including isolates from diarrhoea patients and healthy controls. The EAEC prototype strain 042 was included as positive control (Table S1). As many of the ST31 and ST40 strains were classified as EAEC by PCR probes only, all isolates were first tested for AA on Hep-2 cells which is the current gold-standard for the definition of the EAEC pathotype^5^. As shown in Fig. 1 (images shown for four strains of each ST only), all strains adhered in stacked-brick like aggregates to the cells and underlying coverslips. We further investigated adherence of the EAEC strains to human colonic epithelium *in vitro* and *ex vivo*. On confluent T84 colon carcinoma cells, adhesion of ST40 strains was significantly higher compared to that of ST31 isolates (Fig. 2A). In contrast, no differences in the number of cell-bound bacteria between isolates from cases and controls were detected. Quantification of EAEC growth in cell culture medium alone demonstrated replication of all strains during the 2 h incubation period which did not differ significantly between strains of ST31 and ST40 (Fig. S1). Similar results to those on T84 cells were obtained using IVOC of human colonic biopsies, although colonization levels of ST31 strains were more variable compared to those in cell culture. While all EAEC strains demonstrated AA to colonic tissue, colonization was significantly higher in EAEC strains of ST40 than ST31 (Fig. 2B,C). In addition, ST31 strains from controls adhered significantly better than ST31 isolates from patients with diarrhoea (Fig. 2B, P < 0.01).

### Growth in mucus-containing medium is similar in ST31 and ST40 strains

**Figure 3:**
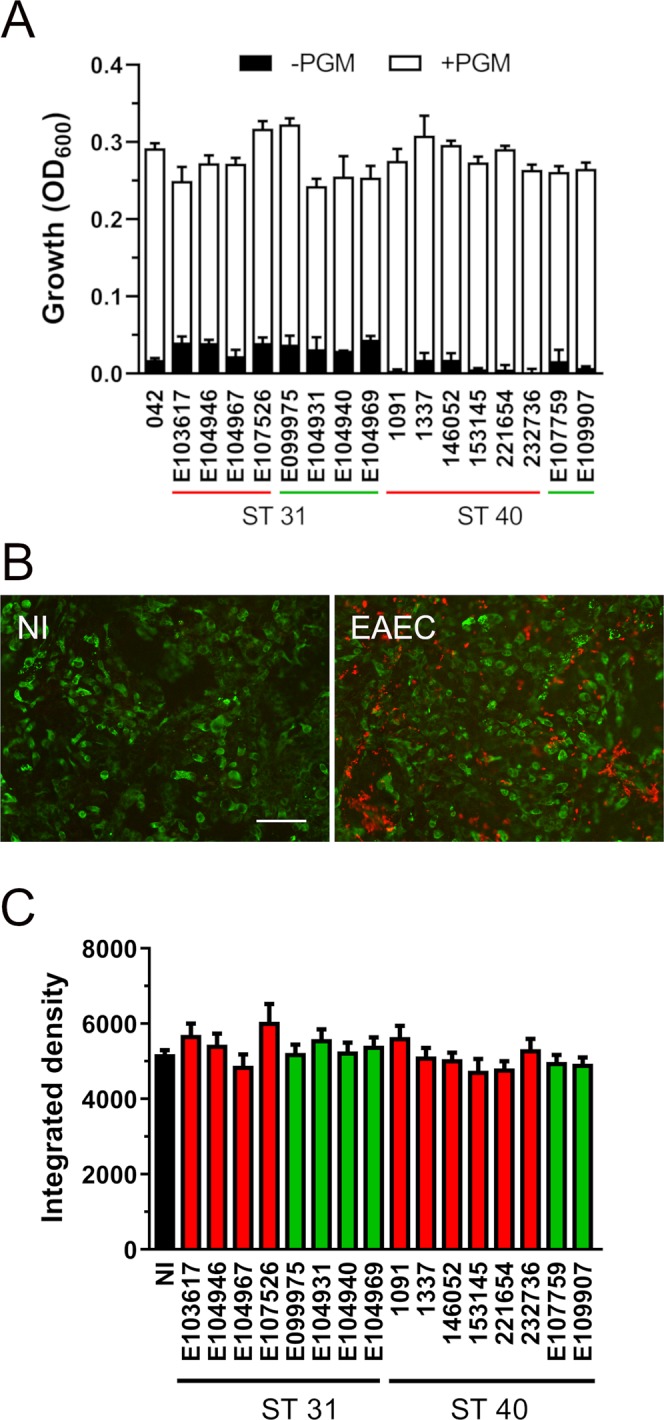
(**A**) Growth of EAEC ST strains in M9 medium with (+PGM) or without 0.5% porcine gastric mucin (-PGM) for 8 h was determined by optical density (OD_600_). Strains from cases or controls are underlined in red and green, respectively. Results represent the mean ± SE from three independent experiments. Data from groups of ST31 and ST40 isolates were analysed using Student’s unpaired t-test. (**B**) Influence of EAEC infection on mucin production by LS174T cells. Cells were infected with EAEC strains for 4 h or left non-infected (NI). Mucus production was visualised by immunofluorescence staining for MUC2 (green), and EAEC were stained in red. Shown are representative images of NI samples and cells infected with E104931 (EAEC). Bar = 50 μm. (**C**) MUC2 staining was quantified by integrated density. Results represent the mean ± SE from at least four independent experiments in duplicate. Data from groups of ST31 and ST40 isolates were analysed using Student’s unpaired t-test.

Previous studies have suggested that utilisation of mucin as a nutrient source by EAEC might provide an advantage for intestinal colonization^21^. We therefore determined the growth of EAEC ST31 and ST40 strains in minimal M9 medium with or without porcine gastric mucin. Growth in M9 alone was low for all strains tested with significantly higher optical densities of ST31 versus ST40 strains (p = 0.017, Fig. 3A). However, addition of mucin enhanced growth of ST31 and ST40 strains to similar levels (Fig. 3A). No significant differences were detected in strains from cases and controls.

### Infection with EAEC ST31 and ST40 strains does not affect mucin levels in LS174T cells

Mucus secretion is a major feature in EAEC-mediated diarrhoea. To investigate the influence of EAEC infection on cellular mucus levels, mucin-producing LS174T cells were infected with EAEC, and production of the major secreted intestinal mucin MUC2 was determined by immunofluorescence staining (Fig. 3B). Subsequent densitometric analysis did not reveal any significant effect of EAEC infection on MUC2 production for any of the ST strains tested (Fig. 3C).

### EAEC ST40 strains form enhanced biofilms compared to ST31 isolates

**Figure 4:**
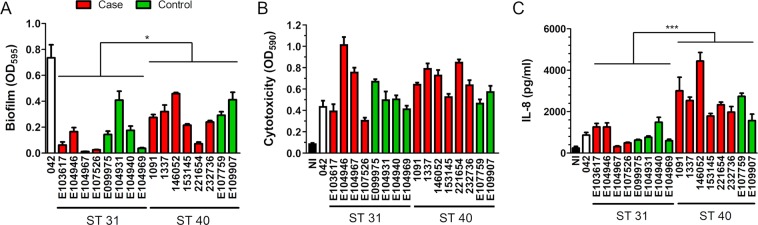
(**A**) Biofilm formation of EAEC ST strains. Bacteria were incubated in 96-well plates for 48 h, stained with crystal violet and absorbance was quantified (OD_595_). (**B**) Cytotoxicity of EAEC infection. Death of T84 cells was quantified by Trypan Blue staining after 8 h of infection. Non-infected cells (NI) were included as controls. (**C**) Secretion of interleukin-8 (IL-8) by T84 cells infected with EAEC. IL-8 levels in supernatants were quantified by ELISA after 24 h of incubation. Results represent the mean ± SE from three independent experiments in duplicate. Data from groups of ST31 and ST40 isolates were analysed using Student’s unpaired t-test. P* < 0.05, P*** < 0.001.

EAEC infection is associated with the formation of thick aggregating biofilms in the mucus layer and at the epithelial surface which impedes antibiotic penetration and efficacy of treatment^22,23^. Interestingly, ST40 isolates demonstrated significantly enhanced biofilm formation on plastic surfaces compared to ST31 strains whilst reference strain 042 demonstrated the highest degree of biofilm formation (Figs. 4A and S2). No difference in biofilm intensity was detected between strains isolated from cases of diarrhoea and healthy controls within each ST.

### EAEC ST31 and ST40 isolates exhibit similar cytotoxicity in T84 cells

A prominent feature of EAEC pathogenesis is the release of cytotoxins which promote epithelial cell death and mucosal damage^6,24^. To determine the cytotoxic effect of ST31 and ST40 isolates on T84 cells, infections were performed for 8 h, and cell death was quantified by Trypan Blue stain. Infection with neither EAEC strain resulted in host cell detachment, and monolayer integrity after staining was confirmed by microscopy (Fig. S3, representative images shown for 042 and the highly cytotoxic strains E104946 and 221654). As shown in Fig. 4B, variable levels of cytotoxicity were observed, particularly among ST31 strains. However, there was no significant difference between the overall levels of T84 cell death caused by ST31 versus ST40 strains or between strains derived from cases or controls.

### ST40 isolates induce higher levels of IL-8 secretion from T84 cells

To evaluate the inflammatory response associated with infection, confluent T84 cells were incubated with EAEC for 3 h. IL-8 concentrations in the supernatant were quantified by ELISA after an additional incubation of 20 h in gentamicin-containing medium, which allows host cell protein synthesis and secretion but prevents bacterial overgrowth. Trypan Blue staining of cell monolayers at the end of incubation confirmed that no significant cell death was caused by any of the strains compared to non-infected controls (data not shown). As demonstrated in Fig. 4C, infection with ST40 isolates resulted in significantly higher levels of IL-8 secretion compared to ST31 strains. In contrast, no association was observed between IL-8 concentrations and whether strains originated from cases or controls

### ST31 and 40 strains exhibit differences in putative virulence genes

**Figure 5:**
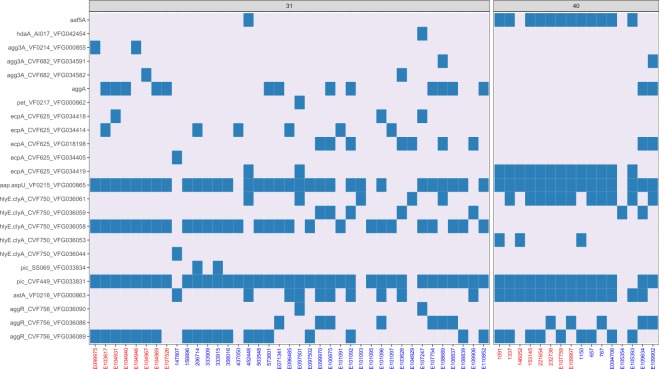
Presence of EAEC virulence-related gene alleles in whole genome sequences of 39 ST31 and 16 ST40 isolates. Strains characterised phenotypically in this study are highlighted in red. Several alleles present in the VFDB were detected for *aggR*, *pic*, *hlyE*, *ecpA*, and *agg3A*, and the presence of each allele is indicated by a blue box. Gene presence was determined by SRST2, except for *aggA* where only a partial read alignment was indicated. In this case, BLAST analysis was used which showed an average nucleotide identity of 86.5%.

To determine genomic differences between the two STs which could explain their different phenotypic properties, whole genome sequencing was performed for all strains. Firstly, we determined the ST of all strains from the sequence reads and confirmed their previously established MLST^20^. In addition, short read sequence typing was used to predict the serotype of each strain in silico which had only been partially established by serum agglutination testing before (Table 1). While ST31 strains belonged to serogroups O130:H27 or O15:H18, ST40 strains mainly displayed O111:H21. There was no considerable difference between serotypes of case or control strains, although more isolates need to be analysed to confirm this. Sequence analysis for EAEC-associated putative virulence genes obtained from GenBank (*aggR* FN554767.1, *pic* FN554766.1, *aap* FN554767.1, *astA* FN554767.1, *hlyE* FN554766.1, *pet* FN554767.1, *ecpA* FN554766.1, *aggA* U12894.1, *aafA* FN554767.1, *agg3A* AF411067.1, *hdaA* EU637023.1, and *aaf5A* AB571097.1) demonstrated that *astA* (EAST-1 toxin) was present in all ST40 but lacking in ST31 isolates (Table 2). Furthermore, *ecpA* (*E. coli* common pilus) was strongly associated with ST40 and encoded by only a quarter of ST31 strains. ST31 and 40 strains also differed in AAF type with ST40 strains harbouring *aaf5A* (AAF/V) and ST31 O130 and O15 strains expressing *aggA* (AAF/I) and *agg3A* (AAF/III), respectively (Table 2). While a coverage threshold of 90% was applied for all read alignments, this was reduced to 80% for *aggA*. In contrast to differences between STs, virulence gene profiles were similar in isolates from cases or controls within each ST. Further differences between ST31 and ST40 were identified using the Virulence Factors of Bacterial Pathogens database (VFDB), and genes found in specific STs only are listed in Table 3. While VFDB analysis confirmed the association of AAF/I and AAF/III with ST31 and the prevalence of EAST1 in ST40 strains, other ST40-specific fimbriae and adhesins (*cfa, ehaA, stg, ycb*) were identified which may contribute to the enhanced host cell binding of this ST. In addition to the 16 strains phenotypically characterised in this study, a further 31 ST31 and 8 ST40 clinical EAEC isolates from PHE were sequenced and analysed. Similar to our previous results, O130:H27 was the predominant serotype in ST31 strains (16 out of 31 strains, Table S2). In addition, three strains were identified as O25:H2, while two isolates belonged to serotype O15:H18 identified in ST31 previously. For ST40, half of the strains encoded O111:H21 (Table S2). Analysis of all ST31 and ST40 whole genome sequences for EAEC-specific virulence gene alleles obtained from the VFDB confirmed the prevalence of *astA* in ST40 (88%) versus ST31 strains (26%, Fig. 5). In addition, all but one ST40 strain harboured *ecpA*, while this gene was present in only 49% of ST31 isolates. Interestingly, one particular allele (*ecpA*_CVF625_VFG034419) was detected in 81% of ST40 versus only 5% of ST31 strains. With regards to AAF, *aaf5A* was the prominent variant in ST40 (75%), whereas more than half of ST31 strains did not harbour any AAF type, and one third were positive for *aggA*. Another interesting finding emerging from the VFDB analysis was that although all ST31 and ST40 isolates possessed a gene for haemolysin E (*hlyE*), different alleles dominated in ST31 (VFG036058) and ST40 strains (VFG036061, Fig. 5).

**Table 1:** EAEC serotype determination.

ST	Strain^a^	Serum agglutination	*In silico* typing
31	**042**	O44:H18	O44:H18
31	E099975	O15:H18	O15:H18
31	E104931	UD	O130:H27
31	E104940	O130:H27	O130:H27
31	E104969	O130:H25	O130:H27
31	**E104946**	UD	O15:H18
31	**E104967**	UD	O15:H18
31	**E103617**	O130:H27	O130:H27
31	**E107526**	UD	O130:H27
40	E107759	UD	O127:H21
40	E109907	O111ab:H-	O111:H21
40	**1091**	O111ac:H11	O111:H21
40	**1337**	O111ac:H11	O111:H21
40	**146052**	0154:H21	O25:H21
40	**153145**	O111:H21	O111:H21
40	**221654**	O111:H21	O111:H21
40	**232736**	O111:H21	O111:H21

^a^Isolates from cases (bold) and controls.

UD = undetermined.

**Table 2:** Detection of selected EAEC virulence genes in whole genome sequences of ST strains by using the Short Read Sequence Typing 2 tool (SRST2).

	Strain^a^	Serotype	*aggR*^b^	*pic*	*aap*	*astA*	*hlyE*	*pet*	*ecpA*	*aggA* (AAF/I)^c^	*aafA* (AAF/II)	*agg3A* (AAF/III)	*hdaA* (AAF/IV)	*aaf5A* (AAF/V)
ST31	**042**	**O44:H18**	+	+	+	+	+	+	+		+			
	**E103617**	**O130:H27**	+	+	+		+		+	+				
	**E104946**	**O15:H18**	+	+	+		+					+		
	**E104967**	**O15:H18**	+	+	+		+					+		
	**E107526**	**O?:H27**		+	+		+			+				
	E099975	O15:H18	+	+	+		+					+		
	E104931	O130:H27	+	+	+		+		+	+				
	E104940	O130:H27	+	+	+		+			+				
	E104969	O130:H27	+	+	+		+			+				
ST40	**1091**	**O111:H21**	+	+	+	+	+		+					+
	**1337**	**O111:H21**	+	+	+	+	+		+					+
	**146052**	**O?:H21**		+	+	+	+		+					
	**153145**	**O111:H21**	+	+	+	+	+		+					+
	**221654**	**O111:H21**	+	+	+	+	+		+					+
	**232736**	**O111:H21**	+	+	+	+	+		+					+
	E107759	O127:H21	+	+	+	+	+		+					+
	E109907	O111:H21	+	+	+	+	+		+					+

^a^Isolates from cases (bold) and controls, ^b^Presence of gene indicated by +,

^c^Coverage threshold below 90% identity.

**Table 3:** Prevalence of bacterial virulence genes in ST31 and ST40 strains as determined from whole genome sequences using Virulence Factor Database.

Gene	Present in all strains of	Function
*EC042_4532*	ST31	Putative type VI secretion protein
*air/eaeX*	ST31	Enteroaggregative immunoglobulin-repeat protein with predicted homology to intimin from enteropathogenic *E. coli*
*aslA*	ST31	Putative Ser-type periplasmic non-aryl sulfatase
*chuA, S-Y*	ST31	Haem utilisation operon
*ehaB*	ST31	Autotransporter linked to adhesion and biofilm formation
*kpsD*	ST31	Polysialic acid transport protein
*pkgA*	ST31	Putative type III secretion effector
*sitA-D*	ST31	Iron/manganese transport operon
*aggB-D*	ST31 (O130)	AAF/I accessory genes
*gspC-M*	ST31 (O130) & ST40	Cryptic type 2 secretion system
*agg3A*	ST31 (O15:H18)	AAF/III structural subunit
*hlyA-D*	ST31 (O15:H18)	Alpha-haemolysin
*papA-K, X*	ST31 (O15:H18)	P pilin operon
*agg3 B-D*	ST31 (O15:H18) & ST40	AAF/III accessory genes
*astA*	ST40	EAST-1 toxin
*cfaA-D*	ST40	CS1 fimbrial operon
*ehaA*	ST40	Autotransporter linked to adhesion and biofilm formation
*stgC,D*	ST40	Part of Stg fimbrial operon
*ycbF,R-V*	ST40	Putative chaperone-usher fimbrial operon

## Discussion

Clinical pathology and laboratory research suggest that EAEC pathogenesis involves adherence to the intestinal mucosa, mucus secretion, biofilm formation, cytotoxic damage and mucosal inflammation due to cytokine release^25^. Importantly, the majority of EAEC *in vitro* and *in vivo* studies have been conducted using prototype strain 042 which we have included as positive control in all phenotypic assays. Although 042 belongs to ST31, it does not share any characteristics with the other ST31 strains investigated in this study. This may be due to differences in origin (Peru versus UK) or changes in genotype/phenotype during prolonged laboratory passage and underlines the heterogeneity of the EAEC pathotype and necessity to open up research to a wider range of EAEC isolates.

Adhesion to the intestinal mucosa is the first step in EAEC colonization of the human gut^22,26^, and increased adherence of ST40 versus ST31 strains could explain the higher association of this ST with diarrhoeal disease. While AA to cervix-derived HEp-2/HeLa cells is supported by all five variants of AAFs^8–12^, a role for binding to human intestinal epithelium has only been demonstrated for AAF/I and AAF/II^8,27^ while the relevance of AAF/III-V remains unknown. Genotypic analysis showed that the ST40 strains investigated in our study possessed the fimbrial variant AAF/V whereas ST31 strains harboured the gene for AAF/I or AAF/III. Notably, alignment of *aggA* (encoding the major pilin subunit of AAF/I) to the query sequence of EAEC strain 17-2 showed less than 90% identity suggesting the presence of a different fimbrial variant. However, high variability with up to 83% identity has been previously reported for the major pilin subunit Agg5A^12^. Furthermore, the presence of the AAF/I accessory genes *aggB-D* in respective ST31 strains supports the identification of AAF/I. Interestingly, expression of AAF/V in *E. coli* HB101 resulted in higher adhesion to HEp-2 cells compared to expression of AAF/III^12^ suggesting different binding affinities of AAF variants to epithelial cell receptors. Previous studies have identified the mucin glycoprotein MUC1, intermediate filament-associated cytokeratin 8, and the extracellular matrix proteins fibronectin, laminin, and type IV collagen as cellular binding partners for AAF/II^28–30^. While AAF/I and AAF/II bind to fibronectin by electrostatic interactions, AAF/V does not recognize fibronectin or other extracellular matrix proteins^31,32^. This might indicate a preference for more accessible host receptors on the apical cell surface (e.g. MUC1) which facilitate initial binding to the intestinal epithelium. Notably, more than half of the ST31 strains sequenced in our study did not harbour any AAF variant, and it remains to be investigated if this contributes to the lower disease potential of this clonal complex. Apart from AAF, other adhesins have been implicated in EAEC - host cell binding. A previous study characterising 130 EAEC strains of diverse origin reported that only 3.1% and 5.4% of isolates were positive for AAF/I and AAF/II genes, respectively, while 96% contained the ECP structural gene *ecpA*, and 63% of those produced ECP when adhering to HEp-2 cells^16^. In addition, an *ecpA* deletion mutant in enterohaemorrhagic *E. coli* was deficient in HEp-2 cell binding. Therefore, the prevalence of *ecpA* and particularly, its specific allele VFG034419, in ST40 versus ST31 strains might suggest a role of ECP in adherence to the gut epithelium. In addition to AAF/V and ECP, other ST40-specific adhesins were identified which include CFA/I fimbriae mediating adherence of enterotoxigenic *E. coli* to human intestinal mucosa^1^, Stg fimbriae promoting epithelial attachment of *Salmonella* Typhi^33^, and the type V secretion system autotransporter EhaA involved in enterohaemorrhagic *E. coli* aggregation and adhesion to bovine rectal epithelial cells^34^.

In addition to epithelial adherence, EAEC interaction with mucus appears to play an important role in infection as evidenced by the presence of mucus in stools of diarrhoeal patients and the thick bacterial biofilms observed in the intestinal mucus layer^6^. Previous studies have shown that the serine protease Pic exhibits mucinolytic activity and enables EAEC growth in mouse caecal mucus and minimal medium containing bovine submaxillary mucin^17,21^. This agrees with our results demonstrating that both EAEC ST31 and ST40 harbour *pic* and can use porcine gastric mucin as a nutrient source. In our previous studies, we have demonstrated that the metalloprotease StcE degrades MUC2 and thereby facilitates adherence of enterohaemorrhagic *E. coli* to human LS174T cells and colonic biopsy samples^35^. However, we did not detect any influence of EAEC infection on MUC2 production by LS174T cells in this study. In addition to its mucinolytic activity, Pic also acts as a secretagogue and stimulates mucus secretion in rat ileal loops^18^. As this is mediated by an increase in goblet cell numbers rather than increased mucin expression by existing goblet cells, an *in vivo* system would be required to investigate this effect.

Mucus secretion facilitates biofilm formation on intestinal mucosa which is a characteristic virulence feature of EAEC^24,36^. Previous studies have shown that AAF/I-V are required for biofilm formation in several EAEC strains including prototype strain 042^9,12,37^. In addition, there may be accessory factors modulating biofilm composition and density as the gene *shf* has been shown to be required for the establishment of firm biofilms by EAEC 042^38^. Interestingly, type 1 fimbriae and flagella which have been implicated in biofilm formation by uropathogenic *E. coli* and *E. coli* K-12^39,40^, did not affect aggregation of EAEC 042^37^. Our results demonstrated increased biofilm formation by EAEC ST40 versus ST31 strains, although variation within ST groups was more pronounced compared to that observed in adherence assays. Similar to cell adhesion, it could be argued that biofilm formation might be dependent on AAF variant, with AAF/V present in ST40 promoting stronger aggregation than AAF/I or AAF/III in ST31. However, this does not apply to ST31 strain E104931 and ST40 strain 221654 which formed stronger and weaker aggregates than the other ST31 and ST40 isolates, respectively. Therefore, additional factors to AAF must play a role in biofilm formation by these EAEC strains.

Cytotoxicity is another prominent feature of EAEC pathogenesis and has been demonstrated in human intestinal biopsies and T84 cells^24^. Previous studies in our laboratory have shown that EAEC binding to T84 cells induces gene expression of the toxins EAST-1, HlyE and Pet suggesting a role in EAEC virulence^41^. As Pet is absent in all ST31 and ST40 strains characterised in this study, and EAST-1 is expressed in ST40 isolates only, these two toxins are unlikely the cause for the cytotoxic effects observed in T84 monolayers infected with EAEC ST31 and ST40 isolates. In contrast, the pore-forming toxin HlyE is encoded by both ST31 and ST40 strains, although different alleles predominate in each ST, and the contribution of HlyE to epithelial damage warrants further investigation.

Mucosal inflammation also contributes to EAEC pathogenesis, and elevated levels of the pro-inflammatory cytokines IL‐1β and IL‐8 have been detected in stool samples from patients with EAEC diarrhoea^42,43^. Several EAEC proteins have been identified which promote secretion of the neutrophil chemoattractant IL-8 *in vitro*, and varying results have been obtained depending on the cell line used and its differentiation status. While the IL-8 response in non-polarized T84 cells infected with EAEC 042 was mainly dependent on flagellin, AafB (minor pilin protein of AAF/II) also contributed to IL-8 secretion by polarized T84 epithelia^44^. Stimulation of IL-8 secretion by flagella and AafB was further confirmed in HEp-2 and Caco-2 cells^45,46^. Another study showed that deletion of the gene for AafA (major pilin protein of AAF/II) severely reduced IL-8 driven neutrophil transmigration across 042-infected T84 epithelia, whilst deletion of the genes encoding AafB or flagellin did not have any effect^47^. In addition, expression of AAF/I, AAF/III, or AAF/IV induced neutrophil transmigration by other EAEC prototype strains, and the dependency of EAEC-induced inflammation on AAF was confirmed in human intestinal xenograft mice^47^. Our studies demonstrated higher IL-8 secretion by T84 cells infected with ST40 versus ST31 strains which might be caused by differences in AAF variant and/or flagellin type. While ST40 strains expressed AAF/V and H21, ST31 strains encoded either AAF/I and H27 or AAF/III and H18. Differences in pro-inflammatory responses to AAF variants have been demonstrated in human intestinal biopsies with higher IL-8 secretion in tissues infected with AAF/II- versus AAF/I- or AAF/III-expressing EAEC strains^48^. Furthermore, purified AAF/II induced greater neutrophil transmigration across T84 cells than AAF/I when applied with *E. coli* K-12^28^. Similarly, uropathogenic *E. coli* expressing H4 flagella induced higher levels of IL-10 secretion compared to isogenic mutants harbouring H1 and H7 alleles^49^.

In addition to differences in ECP, AAF and flagellin alleles, all ST40 isolates in our phenotypic study contained *astA* which was absent in ST31 strains. This gene encodes the low-molecular-weight EAEC heat-stable enterotoxin 1 (EAST1) which shares significant homology with the heat-stable enterotoxin of enterotoxigenic *E. coli*^50^ and stimulates anion secretion by T84 epithelia^51^. While *astA* was significantly associated with infant diarrhoea in Brazil versus asymptomatic controls^52^, the gene was commonly detected in both clinical and non-clinical EAEC strains in China^53^. Therefore, its relevance as a marker for pathogenic EAEC remains uncertain, and future studies are needed to further establish the role of EAST1 in intestinal disease.

While we established a correlation between certain virulence traits and genes with ST31 and ST40 strains, no difference was observed between isolates from diarrhoea cases and asymptomatic carriers within each group. Notably, only two of the investigated ST40 isolates were derived from healthy controls, so further confirmation is required for this ST. Nevertheless, the lack of correlation between virulence trait and strain origin might be due to differences in host susceptibility which can be influenced by genetic and environmental factors and represent a considerable confounding factor in case-control association studies. Single nucleotide polymorphisms in the promoter region of IL-8 and CD14 and the lactoferrin gene have been linked to increased susceptibility to EAEC infection^54–56^. Another predisposing factor appears to be co-infection with other enteropathogens which is considerably higher in EAEC-positive cases than in asymptomatic controls^57^.

Taken together, our studies suggest that EAEC ST40 strains have a higher intrinsic potential to cause enteric disease due to their enhanced ability to bind to human colonic epithelium, form biofilms and induce pro-inflammatory IL-8 secretion. Differences in AAF and flagellin alleles and/or the presence of EAST1 and ECP might represent the underlying cause for these phenotypes. Future studies will ascertain the suitability of these genotypic and phenotypic markers for identification of highly pathogenic EAEC which will improve diagnosis of diarrhoea. In addition, our findings in conjunction with previous data^45^ implicate mucosal adherence as an efficient target for future antimicrobial and vaccine strategies against EAEC infection.

## Methods

### Bacterial culture and identification

All EAEC strains used in this study are listed in Table S1 and S2. EAEC were defined by *aat* gene/CVD432 probe reaction, presence of *aggR* or aggregative adherence phenotype. For all experiments, strains were grown standing overnight in Lysogeny broth (LB Lennox) at 37 °C.

## Cell culture

Human epithelial HEp-2 cells (ECACC 86030501) and LS174T colon adenocarcinoma cells (ECACC 87060401) were cultured in Dulbecco’s Modified Eagle’s Medium (DMEM, Sigma) with 4 mM L-glutamine, 1% non-essential amino acids and 10% foetal bovine serum (FBS, Sigma). T84 colon carcinoma cells (ECACC 88021101) were maintained in DMEM/F-12 1:1 mixture (Sigma) containing 2.5 mM L-glutamine and 10% FBS and used between passage 46 and 63. All cells were grown at 37 °C in a 5% CO_2_ atmosphere.

### Aggregative adherence to HEp-2 cells

HEp-2 cells were seeded at a density of 1 × 10^5^ cells/well into 24-well plates containing circular coverslips and grown to 60% confluency. Cells were inoculated with 10^7^ CFU of EAEC and incubated at 37 °C in air/5% CO_2_ for 3 h. Coverslips were subsequently washed thrice with phosphate-buffered saline (PBS), fixed in 70% methanol for 15 min and stained with 10% Giemsa Modified Solution (Sigma) for 30 min. Samples were observed under brightfield with a Zeiss Axio Imager M2 Microscope.

### Adherence to T84 cells

T84 cells were seeded at a density of 1.5 × 10^5^ cells/well into 24-well plates and cultured for 6-8 days until fully confluent. Cells were inoculated with 10^7^ CFU of EAEC and incubated at 37 °C in air/5% CO_2_ for 2 h. After removal of non-adherent bacteria by three washes in PBS, cells were lysed in 1% Triton X-100 in PBS for 10 min. Serial dilutions of cell lysates and bacterial inocula were plated on LB agar plates, and CFU were counted the next day.

### Bacterial growth in DMEM/F-12 medium

EAEC overnight cultures adjusted to OD_600_ 1.0 were diluted 1:100 in DMEM/F-12 medium and dispensed into 96-well plates in 100 µl aliquots. After incubation at 37 °C for 2 h, optical density was determined at 600 nm.

### *In vitro* organ culture of human colonic biopsies and scanning electron microscopy

This study was performed with approval from the University of East Anglia Faculty of Medicine and Health Ethics Committee (Ref 2010/11-030). All samples were registered with the Norwich Research Park Biorepository (REC reference 19/BE/0089) and all research was performed in accordance with relevant guidelines and regulations. After obtaining informed consent, biopsy samples from the sigmoid colon were taken from adult patients undergoing routine endoscopy at the Gastroenterology Department of the Norfolk and Norwich University Hospital. Biopsy specimens were orientated with the mucosal surface facing upwards on a foam support and submersed in IVOC medium (DMEM/NCTC-135 medium (1:1), 10% newborn calf serum, 1.1 g/l sodium bicarbonate, 0.5% D-mannose, Sigma). Samples were inoculated with 2.5 × 10^7^ CFU of EAEC or LB medium as negative control. Biopsies were incubated at 37 °C in air/ 5% CO_2_ for 7 h on a rocking platform with medium exchange after 4 and 6 h to prevent bacterial overgrowth. At the end of the incubation, biopsies were washed in PBS to remove non-adherent bacteria and fixed with 2.5% glutaraldehyde in PBS at 4 °C overnight. Specimens were subsequently dehydrated in a graded acetone series, dried using tetramethylsilane (Sigma), mounted on aluminium stubs, sputter-coated with gold (Polaron SC7640, Quorum Technologies) and observed using a JSM 4900 LV scanning electron microscope (JEOL). Bacterial colonization was scored on a relative scale based on size of bacterial aggregates (1 = less than 10; 2 = ca 10-100; 3 = ca 100-1,000; 4 = more than 1,000 bacteria) and frequency (1 = less than 10%, 2 = ca 25%, 3 = ca 50%, 4 = more than 50% bacterial coverage of biopsy surface), and both values were added.

### Growth in mucus-containing medium

EAEC overnight cultures adjusted to OD_600_ 1.0 were diluted 1:100 in 200 μl M9 medium (6.8 g/l Na_2_HPO_4_, 3.0 g/l KH_2_PO_4_, 1.0 g/l NH4Cl, 0.5 g/l NaCl, 0.5 g/l glucose) with or without 0.5% (w/v) porcine gastric mucin (Sigma) and added to a 96-well plate. Plates were incubated at 37 °C for 8 h, and bacterial growth was quantified by optical density (OD_600;_ Benchmark Plus, Bio-Rad).

### Quantification of mucus production in LS174T cells by immunostaining

LS174T cells were seeded at a density of 1.5 × 10^5^ cells/well on circular coverslips placed in 24-well plates and grown for 5 days. Cells were inoculated with 10^7^ CFU of EAEC and incubated at 37 °C in air/5% CO_2_ for 4 h. Non-adherent bacteria were removed by three washes in PBS, and cells were fixed in ice-cold methanol/ acetone (1:1) for 4 min on ice. For immunofluorescence staining, cells were blocked with 0.5% (w/v) bovine serum albumin in PBS for 20 min followed by incubation in mouse anti-MUC2 (1:250, Santa Cruz) for 60 min and detection by Alexa Flour 488-conjugated donkey anti-mouse IgG (1:400, Life Technologies). EAEC were stained with goat anti-*E. coli* (1:400, Abcam) and Alexa Flour 568-conjugated donkey anti-goat IgG (1:400, Life Technologies). Coverslips were mounted in Vectashield (Vector laboratories) and examined with a Zeiss Axio Imager M2 Microscope. MUC2 staining was quantified by integrated density using ImageJ software.

### Biofilm formation

EAEC overnight cultures adjusted to OD_600_ 1.0 were diluted 1:100 in DMEM, and 100 µl of suspension were added to a 96-well plate. After incubation at 37 °C in air/5% CO_2_ for 48 h, media were removed, and wells were washed twice with sterile water. Bacterial biofilms at the bottom of the well were stained with 0.1% crystal violet (w/v) for 10 min. After removal of excess stain with two washes in water, plates were dried, and the crystal violet stain was solubilised in 30% acetic acid and quantified by optical density (OD_595_).

### Cytotoxicity assay

Confluent T84 cells in 24-well plates were inoculated with 2 × 10^7^ CFU of EAEC and incubated at 37 °C in air/5% CO_2_ for 8 h. After removal of the medium, dead cells were stained with 0.05% Trypan Blue for 15 min at 37 °C. Unbound dye was removed by washing with PBS, and monolayer integrity was confirmed by microscopy. Internalised dye was subsequently released by cell lysis in 1% SDS, and absorbance was determined at OD_590_.

### Interleukin-8 secretion

T84 cells were seeded out at a density of 4 × 10^5^ cells/well into 12-well plates and grown for 6 days until fully confluent. Cells were infected with 1.5 × 10^7^ CFU and incubated for 3 h at 37 °C in air/5% CO_2_. After removal of non-adherent bacteria, fresh medium containing 50 μg/ml gentamicin (Sigma) was added, and cells were incubated for another 20 h. IL-8 in supernatants was determined by ELISA (Peprotech) according to the manufacturer’s instructions. In addition, cytotoxicity was quantified by Trypan Blue staining as described above.

### Genome sequencing

Extracted bacterial DNA was converted into Nextera XT libraries according to the library preparation guide (Illumina) for sequencing on an Illumina NextSeq platform. Libraries were quantified using HS dsDNA Qubit assay as per the manufacturer’s instructions. Libraries were pooled in equimolar amounts, denatured, and sequenced on the Illumina NextSeq 500/550 platform using V2 2 × 150 bp chemistry with paired-end protocol. Alternatively, sequencing was undertaken using an Illumina HiSeq 2500 system to produce 100 bp paired-end sequence fragments (Illumina, Cambridge, United Kingdom). Genome sequencing was performed at a minimum average depth of 30 ×. All nucleotide sequences were deposited at DDBJ/EMBL/GenBank under accession number ERA1598958.

### Bioinformatics

Bioinformatic analyses were performed using tools accessed via the MRC Cloud Infrastructure for Microbial Bioinformatics (MRC CLIMB) platform, the University of East Anglia Norwich High performance cluster (UEA-HPC) or local platforms as appropriate. FASTQ paired-end reads were quality trimmed using Trimmomatic 0.36, removing adaptors and low quality or N bases from the leading and trailing 30 bp. The read was scanned with a 4-base sliding window and cut when the average quality per base dropped below 15. If the read length post trimming was less than 36 bp, the read and its pair were discarded. MLST determination was achieved using SRST2 and processed locally or on the Galaxy computational biology platform. SRST2 was also used for *E. coli* serotype prediction and virulence factor genotyping. While short read serotyping analysis employed the EcOH database, *Escherichia* virulence gene sequences were obtained from the Virulence Factors of Bacterial Pathogens database (VFDB; http://www.mgc.ac.cn/cgi-bin/VFs/genus.cgi?Genus=Escherichia) with sequences added from NCBI GenBank as required and pre-clustered with cd-hit at 90% identity.

### Statistics

Statistical analysis was performed using GraphPad Prism software (version 6). A P-value of <0.05 was considered significant.

### Data sharing

All data generated or analysed during this study are included in this published article (and its Supplementary Information files). All nucleotide sequences were deposited at DDBJ/EMBL/GenBank under accession number ERA1598958.

## Supplementary information

Supplementary information.
