# Supplementary material for: Identification and characterisation of enteroaggregative Escherichia coli subtypes associated with human disease
Source: Sci Rep. 2020 May 4;10:7475. doi: 10.1038/s41598-020-64424-3 (PMC7198487; doi:10.1038/s41598-020-64424-3)
Supplement: Supplementary file 1 — Supplementary information. [file 41598_2020_64424_MOESM1_ESM.pdf]

Identification and characterisation of enteroaggregative *Escherichia coli* subtypes associated with human disease

Samuel J. Ellis[1,2], Lisa C. Crossman[3,4], Conor J. McGrath[1,2], Marie A. Chattaway[5], Johanna M. Hölken[1,2], Bernard Brett[6], Leah Bundy[3], Gemma L. Kay[1,2], John Wain[1,2], and Stephanie Schüller[1,2*]

Table S1: Strains used in this study

Strain	Isolated from	ST	Source[a]	Year	Country
042	Case	31	PHE	1985	Peru
E099975	Control	31	IID1	1994	UK
E103617	Case	31	IID1	1994	UK
E104931	Control	31	IID1	1994	UK
E104940	Control	31	IID1	1994	UK
E104946	Case	31	IID1	1994	UK
E104967	Case	31	IID1	1994	UK
E104969	Control	31	IID1	1994	UK
E107526	Case	31	IID1	1994	UK
E107759	Control	40	IID1	1995	UK
E109907	Control	40	IID1	1995	UK
1091	Case	40	IID2	2008	UK
1337	Case	40	IID2	2008	UK
146052	Case	40	PHE	2015	UK
153145	Case	40	PHE	2015	UK
221654	Case	40	PHE	2016	UK
232736	Case	40	PHE	2016	UK

[a] Population-based study of infectious intestinal disease (IID), Public Health England (PHE)

Table S2: Additional ST31 and ST40 strains used for genotypic analysis. All strains were isolated from human faeces in the UK. Serotypes have been determined from sequence data.

Strain	ST	serotype	Source	Year
147807	31	O157:H7	PHE	2015
158896	31	O130:H27	PHE	2015
206714	31	O15:H18	PHE	2016
333909	31	O130:H27	PHE	2017
333915	31	O130:H27	PHE	2017
358016	31	O130:H27	PHE	2017
437050	31	O15:H18	PHE	2017
452448	31	O111:H21	PHE	2017
503548	31	O130:H27	PHE	2018
573601	31	O130:H27	PHE	2018
E071341	31	O130:H27	IID1	1991
E096485	31	O130:H27	IID1	1994
E097501	31	O175:H27	IID1	1994
E097502	31	O130:H27	IID1	1994
E099970	31	O3:H2	IID1	1994
E100875	31	O25:H2	IID1	1994
E101091	31	O130:H27	IID1	1994
E101092	31	O25:H2	IID1	1994
E101093	31	O130:H33	IID1	1994
E101095	31	O130:H27	IID1	1994
E101096	31	O130:H27	IID1	1994
E101097	31	O130:H27	IID1	1994

E103626	31	O176:H33	IID1	1994
E104929	31	O62:H30	IID1	1994
E107247	31	O17:H18	IID1	1994
E107754	31	O130:H27	IID1	1995
E108689	31	O92:H33	IID1	1995
E108837	31	O130:H27	IID1	1995
E108839	31	O130:H27	IID1	1995
E109906	31	O25:H2	IID1	1995
E110852	31	O86:H18	IID1	1995
657	40	O111:H21	IID2	2008
767	40	O111:H21	IID2	2008
1150	40	O111:H21	IID2	NA
E094706	40	O111:H21	IID1	1994
E105354	40	O176:H29	IID1	1994
E105393	40	O154:H21	IID1	1994
E109634	40	O25:H2	IID1	1995
E109902	40	O92:H33	IID1	1995

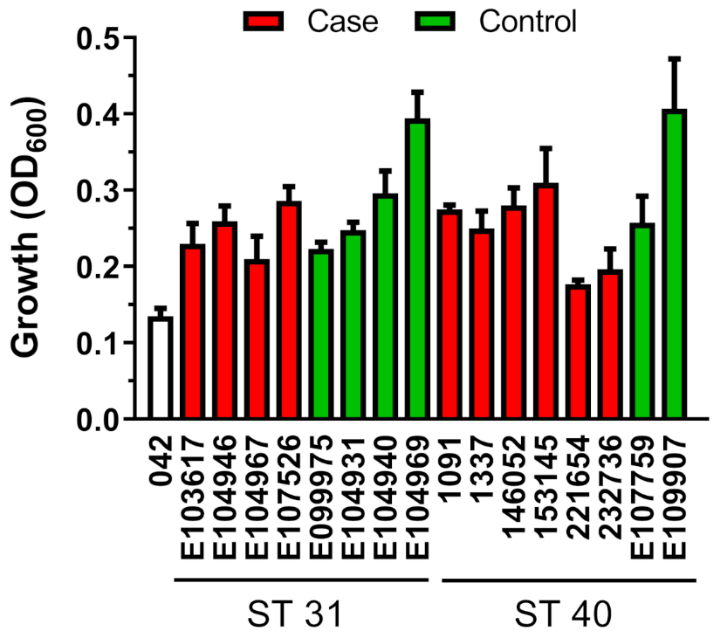

Supplementary Fig. S1: EAEC growth in DMEM/F-12 medium. Bacterial overnight cultures diluted to OD 0.01 in DMEM/F-12 medium were incubated for 2 h. Results represent the mean ± SE from three independent experiments. Data from groups of ST31 and ST40 isolates were analysed using Student's unpaired t-test.

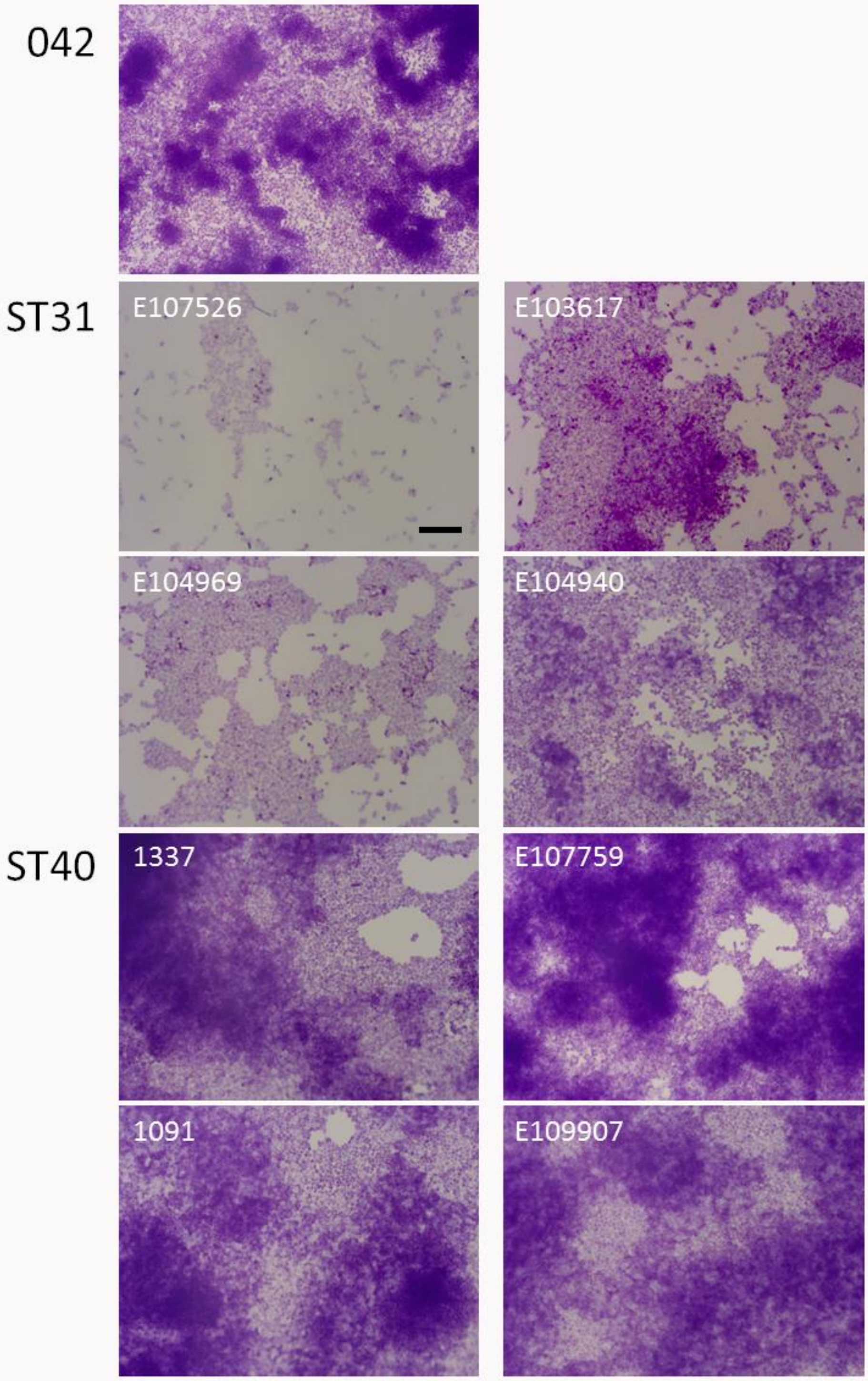

Supplementary Fig. S2: Brightfield microscopy of EAEC biofilms stained with crystal violet. Scale bar = 10 µm.

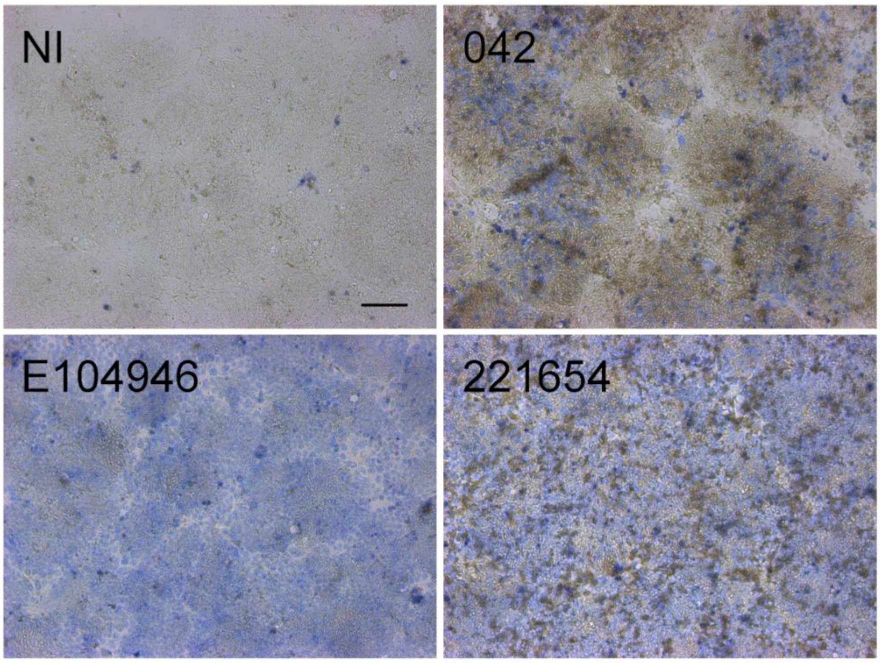

Supplementary Fig. S3: Monolayer preservation after Trypan Blue stain. Confluent T84 cells were infected with EAEC 042, E104946, 221654 or left non-infected (NI) for 8 h. Cells were subsequently stained with Trypan Blue to assess EAEC cytotoxicity. Bar = 50 µm.
